# *De Novo* Transcriptome Sequencing and Analysis for *Venturia inaequalis*, the Devastating Apple Scab Pathogen

**DOI:** 10.1371/journal.pone.0053937

**Published:** 2013-01-17

**Authors:** Karnika Thakur, Vandna Chawla, Shammi Bhatti, Mohit Kumar Swarnkar, Jagdeep Kaur, Ravi Shankar, Gopaljee Jha

**Affiliations:** 1 Biotechnology Division, CSIR-Institute of Himalayan Bioresource Technology (Council of Scientific and Industrial Research), Palampur, Himachal Pradesh, India; 2 Studio of Computational Biology & Bioinformatics, CSIR-Institute of Himalayan Bioresource Technology (Council of Scientific and Industrial Research), Palampur, Himachal Pradesh, India; 3 Department of Biotechnology, Panjab University, Chandigarh, India; University of California Riverside, United States of America

## Abstract

*Venturia inaequalis* is the causal agent of apple scab, one of the most devastating diseases of apple. Due to several distinct features, it has emerged as a model fungal pathogen to study various aspects of hemibiotrophic plant pathogen interactions. The present study reports *de novo* assembling, annotation and characterization of the transcriptome of *V. inaequalis*. *Venturia* transcripts expressed during its growth on laboratory medium and that expressed during its biotrophic stage of infection on apple were sequenced using Illumina RNAseq technology. A total of 94,350,055 reads (50 bp read length) specific to *Venturia* were obtained after filtering. The reads were assembled into 62,061 contigs representing 24,571 unique genes. GO analysis suggested prevalence of genes associated with biological process categories like metabolism, transport and response to stimulus. Genes associated with molecular function like binding, catalytic activities and transferase activities were found in majority. EC and KEGG pathway analyses suggested prevalence of genes encoding kinases, proteases, glycoside hydrolases, cutinases, cytochrome P450 and transcription factors. The study has identified several putative pathogenicity determinants and candidate effectors in *V. inaequalis*. A large number of transcripts encoding membrane transporters were identified and comparative analysis revealed that the number of transporters encoded by *Venturia* is significantly more as compared to that encoded by several other important plant fungal pathogens. Phylogenomics analysis indicated that *V. inaequalis* is closely related to *Pyrenophora tritici-repentis* (the causal organism of tan spot of wheat). In conclusion, the findings from this study provide a better understanding of the biology of the apple scab pathogen and have identified candidate genes/functions required for its pathogenesis. This work lays the foundation for facilitating further research towards understanding this host-pathogen interaction.

## Introduction

*Venturia inaequalis* (Cke.) is a phytopathogenic fungus that causes Apple scab, the black spot disease of apple [1], [2], [3]. It causes deformation in shape and size of the affected fruits, premature leaf/fruit fall and enhances susceptibility of apple tree to chilling and freezing injuries. Overall, it renders apple unsuitable for trade and causes up to 70% of yield reductions. Like other obligate parasites, it generally infects and lives in association with living host tissues. However, its ability to be cultured on laboratory medium, possibility of *in vitro* mating, existence of extensive population diversity, uninucleate conidia, genetically uniform progenies, stability of genotype and phenotype of the progeny even after multiple rounds of sub-culturing and availability of standardized protocol for genetic manipulation, etc. makes it a useful model to study the pathogenesis of hemibiotrophic fungal pathogens [1], [2], [3]. *V. inaequalis* has broad geographical distribution and an interesting growth pattern. It is mostly restricted in sub-cuticular space of the apple tissues, does not form haustoria, and no apparent mechanical pressure is observed during its penetration into host cuticle [1].

*V. inaequalis* is rapidly evolving, hyper variable and eight different races have been reported from different parts of the world [1]. Using multi locus microsatellite markers, Gladieux et al [4] had revealed the origin of *V. inaequalis* in central Asia (which is also centre of origin of apple), from where it seems to have traveled along with apple (domestication/introduction) using silk route to get into Europe and expanded into other continents. Practices like disease resistance breeding and protective fungicide spray are being routinely used to control apple scab disease [3], [5], [6]. Several disease resistance (*R*) genes have been identified in apple and one of them, namely *Rvi6* (*Vf*) has been cloned, which is being explored to engineer scab resistant apples [7], [8]. But both of these commonly utilized methodologies to control apple scab, are facing big threats as the pathogen is rapidly evolving resistance against fungicides [9], [10], [11] and has breached several *R* gene mediated resistance [12], [13], [14]. So far, inadequate studies have been done to understand the molecular interaction between *Venturia* and apple, which could otherwise provide insights to develop alternative strategies to prevent scab disease.

*V. inaequalis* is a heterothallic fungus and contains seven chromosomes. Its genome size is estimated to be around 100 Mb [15] while most of the other ascomycetes genomes are mostly in the range of 40 Mb. The best available genetic map for *V. inaequalis* consists of eleven linkage groups with total map length of 1106 cM [16]. Efforts have been made to identify effectors which might determine the pathogenicity of this pathogen. Recently sixteen putative candidate effectors have been mined from *Venturia* ESTs, three of them were found to be differentially regulated during *in planta* growth [17]. Although the functions of these effectors has not been established as yet. In several other pathosystem, it has been demonstrated that pathogen uses effectors to suppress host immunity, while some of these effectors (better known as avr factors) induces host immunity upon being recognized by the host *R* gene(s). Hence, identifying the *avr* genes and those involved in suppressing host immunity would be useful in ensuring durable scab resistance in apple.

With the introduction of Next Generation Sequencing (NGS) platforms like Roche 454, Illumina Genome Analyzer and Applied Biosystems SOLiD, there is an exponential increase in both genome and transcriptome sequencing efforts [18], [19], [20], [21]. Advancement of various *de novo* sequence assembly algorithms has made it possible to assemble short reads obtained by these NGS platforms to generate draft genome/transcriptome sequences [22], [23], [24]. These tools are being utilized to understand the intricacies of host-pathogen interactions by unraveling the genomic and transcriptome sequences. Transcriptome sequencing is becoming an economically attractive alternative to whole genome sequencing, as one can study the expressed part of the genome and bring novel insights about the underlying biological processes. In the present study, Illumina Genome analyser *GA IIx* has been used to sequence the transcriptome of *V. inaequalis* and obtain genomic insights about this devastating pathogen. A total of 94,350,055 reads of *V. inaequalis* transcriptome, assembled into 62,061 contigs, representing 24,571 non-redundant unigenes, were functionally annotated. The present work also predicts the secretome, effectors and genes involved in host-pathogen interactions. Also the phylogenomic study of *Venturia* with respect to several ascomycetes fungal pathogens have been explored in this study.

## Materials and Methods

### Isolation, culturing and bioassay of *Venturia inaequalis*

Indian isolate of *V. inaequalis* was isolated from the diseased apple fruit sample collected in a sealed polyethylene biohazard bag, from Kullu district of Himachal Pradesh, India (No permission was required to collect the diseased apple fruit sample to carry out this research work). Dilution plating and repeated sub-culturing on PDA (39 g/L; Potato Dextrose Agar; HiMedia, Mumbai, India; plates incubated at 20°C) were used to obtain pure culture of *V. inaequalis*. The identity of the pathogen was confirmed through microscopic analysis, rDNA sequencing and by testing its ability to cause scab symptoms on susceptible cultivar Gala. In order to enrich the transcriptome, we explored the transcripts expressed during its growth on apple and on laboratory media. 10 µl mycelial suspension isolated from one month old culture of *Venturia* (grown on PDA plate; at 20°C) was inoculated on the detached leaves of susceptible apple cultivar Gala as described by Win et al. [25] and also in 2 ml of PDB (24 g/L; Potato Dextrose Broth; HiMedia, Mumbai, India) broth with proper shaking. To prevent microbial contaminations generally associated with field grown apple leaves (which might potentially create confusion during assembling *Venturia* specific transcripts), we used *in vitro* grown leaves of Gala in this study. The samples were collected from both the *Venturia* inoculated leaves and *Venturia* growing in PDB broth at 0, 2 and 5 dpi (day post inoculation).

### Library preparation and transcriptome sequencing

Total RNA from each of the collected samples was isolated using iRIS [26]. The integrity and quantity of isolated RNA were assessed on Bioanalyser (Agilent; 2100). Further, 4 µg RNA from each of these samples was processed for RNA library preparation using TruSeq RNA sample Prep Kit (Illumina) as per manufacturer's instruction. Library was quantified using Qubit™ RNA assay kit for Qubit 2.0® Fluorometer (Life technologies) and 10 pM of the prepared library were loaded onto a flowcell for cluster generation. The following loading arrangement was used: Lane 2: *Venturia* (0 dpi), Lane 3: Gala leaves infected with *Venturia* (2 dpi), Lane 4: PhiX Control, Lane 5: PDB grown *Venturia* (2 dpi), Lane 6: Gala leaves infected with *Venturia* (5 dpi), Lane 7: PDB grown *Venturia* (5 dpi), Lane 8: mixture of all above mentioned libraries except PhiX control. As per our previous experience, we selected 10 pM concentration of prepared library for cluster generation in this study and obtained good number (∼470–540 k/mm^2^) of clusters. Library sequencing was performed on Illumina Genome Analyzer *GAIIx* as per manufacturer's instructions (Illumina Inc).

### *De novo* assembly and sequence clustering

The raw sequencing data was transformed into Single End (SE) 72 bp reads, using GERALD base-calling (a CASAVA package tool provided by Illumina). Resulting sequence reads were stored in FASTQ format. The 3′-end read trimming (keeping first 50 bp) was performed using read filtering tool, filteR [27]. The reads for *V. inaequalis* were obtained from different lanes of the flowcell. Three lanes (lane 2, 5 and 7) were having only *Venturia* specific transcripts (as they were covering different stages of its growth on laboratory medium). Other three lanes (lane 3, 6 and 8) were containing mixed sample for *Venturia* and apple (as they were processed either from *Venturia* infected apple leaves: lane 3 and 6; or from the mixture: lane 8). To remove apple specific reads (from lane 3, 6 and 8), reads were mapped onto apple genome [28] using Bowtie [29] with default settings. The unmapped reads were considered to have come from *V. inaequalis*. *De novo* assembling of high quality reads was performed using SOAPdenovo, ABySS and Velvet and in combination with a series of stepwise strategies [22], [23], [24], [27], [30]. The best assembled transcripts from each assembler (SOAPdenovo, ABySS and Velvet) were selected; reflecting the best balance between the number of contigs produced, average coverage, N50 length value of total assembly and average sequence length attained. Redundant set of sequences were removed by filtering and merging similar sequence stretches with TGICL-CAP3 [31] and CD-HIT-EST [32] based sequential and hierarchical clustering.

### Assembly validation and similarity search for assembled *V. inaequalis* transcripts

**Figure 1:**
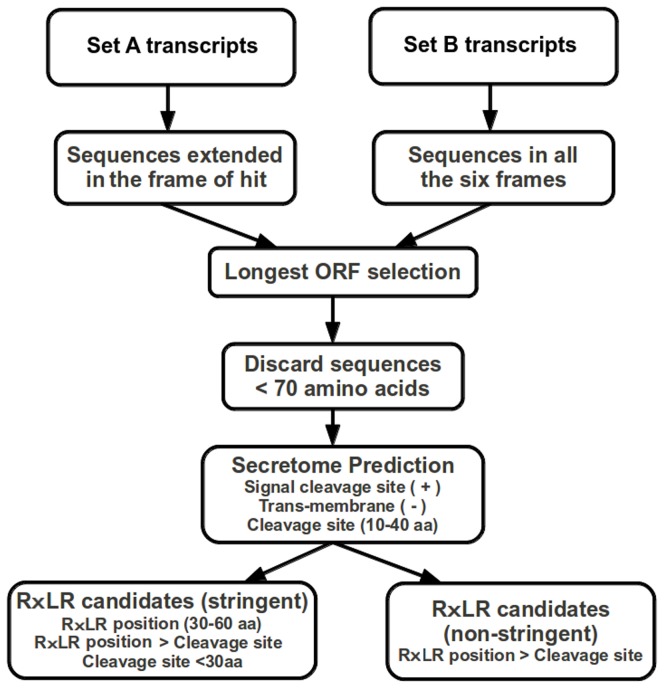
Workflow of RxLR effector prediction. A flow chart describing the strategies for prediction of RxLR effectors and open reading frame of the transcripts.

To assess the reliability of assembly, 155 experimentally validated nucleotide sequences for *V. inaequalis* available at GenBank were used **(File S1)**. BLASTn analysis was performed for reported nucleotide sequences against the set of assembled transcripts sequences at E-value threshold of 1e^−05^. Primer pairs were designed to amplify the partial sequences of 15 randomly selected transcripts and the PCR amplicons were sequenced using dye Terminator 3.1 Cycle Sequencing Kit (Applied Biosystems Foster City,USA) on 3130 XL Genetic analyzer from Applied Biosystem. BLAST2 analyses were performed to determine the homology of the sequences obtained through Sanger sequencing with that obtained through *de novo* assembly of the transcriptome. For similarity search, the assembled and filtered transcript sequences obtained after hierarchical clustering were scanned against NR protein sequence database [33] using BLASTx [34] with E-value threshold of 1e^−05^. Further, these assembled transcripts were clustered on the basis of BLASTx hits against NR database. Several assembled transcript sequences, though display absolutely no similarity with each other, but map to the different parts of the same gene. Considering this, such transcripts were grouped into a single group representing unique genes. This reduces the inflated number of transcripts and gives better representation of total unique genes identified. The above strategy is known as Dissimilar Sequences (DS) clustering [27] and the same has been adopted in the present study to obtain more precise transcriptome representation for *V. inaequalis*. The non-redundant *V. inaequalis* transcripts obtained after DS clustering are referred to as Set A transcripts, while the transcripts with no homology in BLAST analysis have been referred to as Set B transcripts. The transcripts in Set A and Set B were translated as described in **Figure 1**.

### Sequence annotation and protein family classification

Assembled transcripts of *V. inaequalis* were searched against UniProt database [35] to assign associated GO [36], KEGG [37], [38] and EC [39] based annotation using Annot8r annotation tool [40], with E-value threshold of 1e^−01^. Protein families were classified by searching the assembled transcripts against Pfam [41] and InterProScan [42]. Pfam database was searched using HMMER3 [43] and the Conserved Domain database [44] was scanned using RPS-BLAST [45]. Protease families were identified using BLASTp (E-value <10^−20^) against MEROPS peptidase database release 9.6 [46]. Cytochromes (CYPs) were named according to classification details collected from BLASTp (E-value <10^−5^) against Fungal Cytochrome P450 database version 1.2 [47]. Transporters were classified based on BLASTp (E-value <10^−10^) against Transporter Classification Database [48]. KinBase database was scanned using BLASTp (E-value <10^−10^) to characterize sequences belonging to Kinase families [49]. Carbohydrate-degrading enzymes selected from InterProScan and Pfam analysis were classified according to GH (Glycoside hydrolase) family as classified in CAZy database [50]. PHI-base (pathogen-host interaction database) [51] was searched for different phenotypic categories like loss of pathogenicity, reduced virulence, effector, lethal, increased virulence etc. with BLASTp (E-value <10^−5^). As Set B transcripts did not show any homology while performing BLAST searches, only Set A transcripts of *Venturia* were used for comparative analysis. Proteases, carbohydrate-degrading enzymes and membrane transporters were predicted from *Venturia* transcriptome and compared with that encoded by *Magnaporthe oryzae*, *Fusarium graminearum*, *Botrytis cinerea* and *Sclerotinia sclerotiorum*. The dataset for proteases and membrane transporters were obtained from Zheng et al [52] while the carbohydrate-degrading enzymes were obtained from CAZy [50].

### Secretome and RxLR effector identification

The amino acid sequences (Set A and Set B) of *V. inaequalis* transcriptome were further analyzed for prediction of secreted proteins. Sequences smaller than 70 amino acids, were not considered for further analysis (**Figure 1**). The remaining sequences with positive SignalP [53] prediction for signal peptide cleavage site at N-terminal region between 10–40 amino acids, without any transmembrane region as predicted by TMHMM [54], were selected as the candidate secreted proteins. RxLR pattern was searched using FuzzPro, a tool from EMBOSS package [55]. Sequences having RxLR patterns positioned between 30–60 amino acids and appearing after the observed signal cleavage site (30 amino acids from the start) were considered [56]. Also a less stringent approach was applied to predict RxLR effectors, wherein any protein with RxLR pattern and signal peptide cleavage site were considered as candidate RxLR effectors **(** **Figure 1** **)**. The predicted secretome was also searched for similarity across known set of effectors **(File S2, S3)** using BLASTx (E-value <10^−2^). First set contained the sequences of known effectors of phytopathogenic fungi (n = 32) and the second set contained previously reported sequences of *V. inaequalis* predicted effectors (n = 16) [17].

### Phylogenomic analysis

The reference protein sequences of nine species namely, *Aspergillus fumigatus*, *Aspergillus nidulans*, *Candida albicans*, *Magnaporthe oryzae*, *Neurospora crassa*, *Sclerotinia sclerotiorum*, *Pyrenophora tritici-repentis*, *Gibberella zeae*, *Botryotinia fuckeliana*, were downloaded from NCBI Protein RefSeq database [57]. In case of *V. inaequalis*, the amino acid sequences from transcripts with k-mer coverage [24] greater than or equal to 50 were used for ortholog detection and phylogenetic analysis. The sequences were analyzed using Hal, an automated pipeline for phylogenetic analysis of high throughput data [58]. Amino acid sequences of all ten species (*A. fumigatus*, *A. nidulans*, *C. albicans*, *M. oryzae*, *N. crassa*, *S. sclerotiorum*, *P. tritici-repentis*, *G. zeae*, *B. fuckeliana* and *V. inaequalis*) were imported in FASTA format and subjected to all vs all BLASTp search. MCL clustering algorithm [59] with a range of inflation parameters from 1.1 to 5 was used to group amino acid sequences into orthologous clusters. These clusters were screened for any possible redundancy. Redundancy could exist if clusters were found at more than one inflation parameter, clusters contained more than one amino acid sequence per genome, or the clusters containing amino acids sequences whose best reciprocal BLAST hit was found outside the cluster. Amino acid sequences for accepted orthologous clusters were extracted from their respective proteome dataset in FASTA format and subsequently aligned using ClustalW [60]. To counter uneven regions of alignments, three separate super-alignments were created: 1) by removing all gap-containing columns (remgaps), 2) by removing uneven regions of alignments based on the default conservative option (Gblocks-con) and 3) by liberal (Gblocks-lib) options of the Gblocks tool [61]. Best models of amino acid substitution for alignment were determined using ProtTest [62] and by the AIC criterion [63] with 10 models: Dayhoff, Blosum62, JTT, MtREV, WAG, RtREV, CpREV, VT, MtMM and GTR. For analysis, three phylogenetic trees were generated, representing three super-alignments (remgaps, Gblocks-con and Gblocks-lib). RAxML [64] was run on each of the super-alignments with the PROTCAT setting for the rate model with best-fitting model of amino acid substitution. Nodal support was estimated based on 1000 bootstrap replications using *Candida albicans* as an out-group. The trees were visualized using Dendroscope [65].

## Results and Discussion

*V. inaequalis* is one of the most important plant pathogen. Besides being havoc to apple industry, it has an interesting life style [1], [2]. Efforts are being made to understand the genomic structure and genome sequence of this deadly pathogen [16], [66], [67]. Understanding its transcriptome would provide insights about its pathogenicity mechanisms and arsenals being used to invade apple. With the advancement of sequencing tools and techniques, it is now possible to explore the entire transcriptome at a time. In the present study, we have applied Illumina Next Generation Sequencing platform to unravel the transcriptome of an Indian isolate of *V. inaequalis*. The present work reports transcriptome sequencing and *de novo* assembly of obtained short reads, comprehensive annotation of assembled sequences, protein-family classification, effector identification and exploration of phylogenomic relationship of this deadly pathogen with other ascomycetes fungi.

### *De novo* sequence assembly of *V. inaequalis* transcriptome

**Figure 2:**
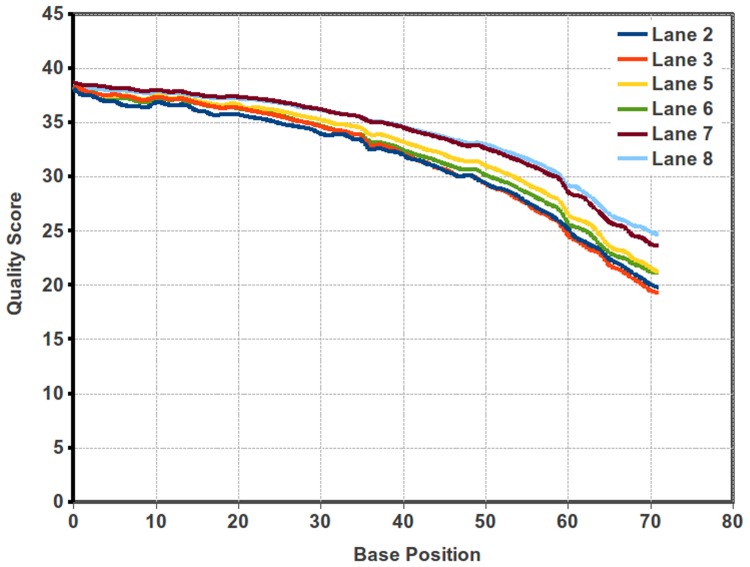
Quality score for each lane of Illumina reads. The line diagram of the quality score of the reads obtained from different lanes.

*De novo* assembly of short reads without a reference genome still remains a challenge, in spite of recent development of several computational tools and approaches for data assembly and analysis. Single-end (SE) run of 72 cycles was performed on Illumina *GAIIx* for different growth/infectious stage of *V. inaequalis*. Quality check through filteR tool revealed comparatively declining read quality at 3′-end **(** **Figure 2** **)**. It was found that the average read quality was acceptable up to 50^th^ cycle. Therefore, trimming of reads was done to maintain read length of 50 bp. This way, a total of 147,780,763 SE reads were generated, out of which 129,766,417 reads passed quality filtering. After removing apple specific reads by mapping them onto apple genome, a total of 94,350,055 reads remained specific for *V. inaequalis* **(** **Table 1** **)**. To obtain transcript sequences, the best assembled transcripts set from different available tools (Velvet, ABySS and SOAPdenovo) at different k-mers ranging from 19 to 47 were selected. The parameters considered were: transcripts having assembly length higher than 100 bp, average coverage, average transcript size, percentage of transcripts having length higher than 1000 bp, N50 value and highest transcript length. K-mer size of 29, in case of Velvet and SOAPdenovo and 27, in case of ABySS, emerged as the best choices for performing assembly. They displayed the best balance between transcripts number, coverage, maximum and average transcript length **(File S4)**. At selected k-mer sizes, a total of 68,027 (with SOAPdenovo), 26,678 (with Velvet) and 45,805 (with ABySS) assembled transcripts were obtained. The average length for these transcript assemblies were 459 (with SOAPdenovo), 815 (with Velvet) and 568 (with ABySS) bases, having average coverage of 71, 170 and 108, respectively. Thus, the combined initial assembling of *V. inaequalis* transcriptome sequences yielded 140,510 transcripts.

**Table 1:** Summary of reads count from different lanes.

Lane	Reads count before filtering	Reads count after filtering	Reads mapped on Apple genome	Reads count used for *Venturia* assembly
2	22,194,292	18,539,123	NIL	18,539,123
3	23,519,220	20,121,283	13,496,583	6,624,700
5	25,668,062	22,565,870	NIL	22,565,870
6	24,606,172	21,176,695	14,401,217	6,775,478
7	24,624,446	22,540,628	NIL	22,540,628
8	27,168,571	24,822,818	7,518,562	17,304,256
Total	147,780,763	129,766,417	35,416,362	94,350,055

### Homology search and sequence clustering

Several sequences shared similarity between the different assembly sets (obtained from different assembling tools as described above) as well as exhibited overlap within each set, causing redundancy. In order to remove such redundancy and overrepresentation of transcripts, sequence similarity based hierarchical clustering was performed to merge such sequences After performing hierarchical clustering with TIGR Gene Indices clustering tools (TGICL), contig assembly program (CAP3) and Cluster database at high identity with tolerance (CD-HIT), number of assembled transcripts got reduced from a total of 140,510 to 62,061. A total of 29,750 such assembled sequences returned significant BLASTx hits while no hit was found for 32,311 sequences. Another clustering step was carried out for sequences with significant BLAST hits. Sequences with no apparent significant identity among themselves might belong to different parts of the same gene or may represent different isoforms. Counting them as separate transcripts would only inflate the number of unique genes [27]. Therefore, all such transcripts which returned hit to some common reference gene were assigned to a common cluster group, representing a unique gene. A set of in house scripts were used to scan for all those assembled transcript sequences that returned best hit with common reference but differed in their location. This step reduced the total number of transcripts with significant BLAST hits, from 29,750 to 24,571 (**File S5**). For the sake of uniformity, here onwards the non-redundant transcripts of *V. inaequalis* with BLAST hits (24,571) are referred to as Set A, while the transcripts with no homology in BLAST analysis (32,311) have been referred to as Set B. It is to be noted that Set B transcripts could also display the above mentioned clustering property and their total number might go lower than the observed value. Assembly statistics at both levels of clustering are given in **Table 2**, revealing average coverage being 71.63 and 127.42 for sequence similarity based clustering (with combination of TGICL and CD-HIT) and DS clustering, respectively. The sequences for Set A and Set B transcripts of *V. inaequalis* are provided in **File S6** and **File S7**, respectively.

**Table 2:** Summary statistics for sequence assembling and clustering.

Assembler	Total Transcripts (> = 100)	Max Length	Average Length	Average coverage	Transcript Length > = 1000	% transcripts (> = 1000)	Total-length (bp)	N50 (bp)
Soap-Denovo	68027	27586	458.58	70.58	8258	12.14	31195796	1418
Velvet	26678	20909	814.96	170.06	7333	27.64	21741489	1979
ABySS	45805	16036	567.59	107.62	7722	16.86	25998311	1445
Sequence similarity based clustering	62061	27669	507.257	71.63	8511	13.71	31480907	1629
Total Unique Gene groups	24571	15576	918.88	127.42	7468	30.39	22577929	2188

### Validation of assembled sequences

The assembled transcriptome sequences were validated by BLASTn search against 155 publically available nucleotide sequences of *V. inaequalis* **(File S1)**. Significant hits were observed for 131 sequences (84.51%), while no hit could be obtained for 24 previously reported nucleotide sequences of *V. inaequalis*. Most of the assembled transcript sequences were found correctly aligning in continuous manner, with an average identity of 98.79. The observed minimum coverage was 8.29% while maximum coverage was found to be 100%. Out of 131 nucleotide sequences, 105 sequences had at least 50% coverage, suggesting overall good assembly quality **(File S8)**. Out of 24 NCBI sequences for which no hit was found across the assembled transcriptome of *V. inaequalis*, 15 were corresponding to 18S ribosomal RNA sequences and 6 were microsatellite sequences. The remaining three sequences for which no hit was found could be some stage specific transcripts. This is to be noted that even in finished genomes like *A. thaliana* around, 13% of the known nucleotide sequences could not be assigned to the final assembly [68]. In case of human only 64% of the reads could be mapped onto the RefSeq database of well annotated human genes [69].

To further validate the *de novo* transcriptome sequence assembly partial fragments of 15 randomly selected transcripts were PCR amplified and sequenced using Sanger dye termination based method. **File S9** summarizes the BLAST2 analysis demonstrating high score, E-value and identity between the sequences obtained through Sanger sequencing with that of *de novo* assembled transcripts sequences.

### Functional annotation and classification of *V. inaequalis* transcriptome

**Figure 3:**
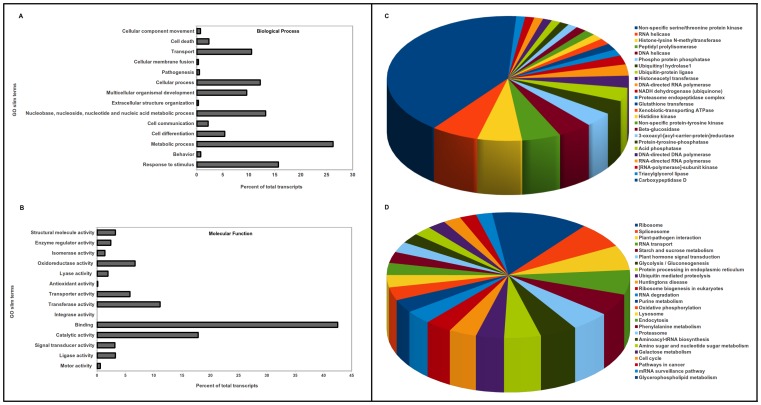
General annotation of *V. inaequalis* transcripts. Gene Ontology (GO) term assignments to *V. inaequalis* unigenes, based on significant GO slims, summarized into two main GO categories: biological process (**A**), and molecular function (**B**). Functional characterization and abundance of *V. inaequalis* transcriptome for enzyme classes, and KEGG pathways are represented in **C and D** respectively. Only top 25 most abundant EC and KEGG pathways are represented. Area under each pie represents the value in percent.

For functional annotation, the *V. inaequalis* transcripts were compared against amino acid sequences available at UniProt database using BLASTx algorithm. The associated hits were searched for their respective Gene Ontology (GO), Kyoto Encyclopedia of Genes and Genomes (KEGG) and Enzyme Commission Codes (EC) for each query sequence and the highest bit score was selected. Annotation against GO database yielded significant hits for 18,431 out of 24,571 unigenes of *V. inaequalis*. **Figure 3A** and **Figure 3B** represent the distribution of *V. inaequalis* transcripts across the various GO categories associated with biological process and molecular function, respectively. Under biological process, categories like metabolic process, response to stimulus, nucleic acid metabolism, cellular process and transport etc were highly represented. For molecular function ontology, genes associated with binding, catalytic, transferase and oxidoreductase activities were found most abundant. This indicates occurrence of rapid growth and extensive metabolic activity for this pathogenic fungi.

**Figure 4:**
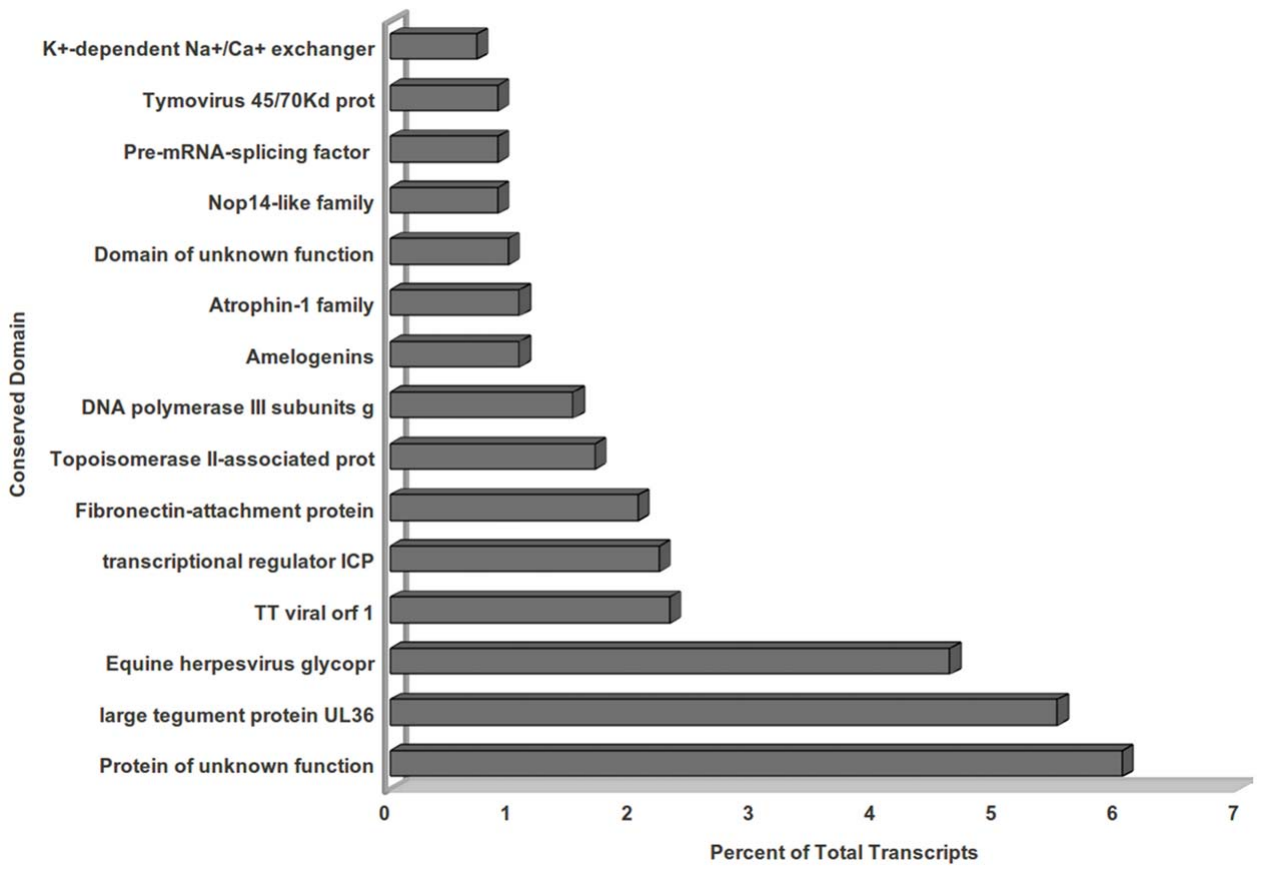
Bar Graph illustrating the Conserved Domains in Set B transcripts. Sequences showing no homology with nr database (Set B transcripts) were subjected to Conserved Domain Database (CDD) to predict the conserved domain. Top 15 highly represented functional conserved domains are shown by bars.

Best EC classification was obtained for a total of 9,731 unique genes, and KEGG classification was obtained for 10,821 unique genes. **Figure 3C** enlists the top 25 abundant enzyme classes observed for *V. inaequalis* unigenes. Interestingly, a large amount of transcripts belonged to nonspecific serine/threonine protein kinase enzyme class (12.82%). **Figure 3D** displays top 25 KEGG pathways represented by *Venturia* unigenes. Large proportion of such transcripts belonged to ribosome (4.84%), spliceosome (2.97%) and pathways involved in plant-pathogen interaction (2.90%). This was followed by RNA transport, starch and sucrose metabolism, plant hormone signal transduction, glycolysis/gluconeogenesis, protein processing in endoplasmic reticulum, ubiquitin mediated proteolysis and others. An InterproScan analysis of unigenes identified 5,418 conserved protein families in *V. inaequalis*, Pfam scan resulted in identification of 3,960 families. As Set B transcripts did not show any significant homology in BLAST analysis, we performed Pfam and InterProScan analysis to obtain insights about their putative functions. The analysis resulted in identification of 475 families with InterproScan and 352 families by Pfam scan. As shown in **Figure 4**, presence of conserved domain in several of Set B transcripts suggests that they might be encoding genes with important functions such as fibronectin attachment protein, topoisomerase, transcriptional regulator, DNA polymerase, pre-mRNA spicing factors etc.

BLASTp analysis against KinBase database resulted into 240 hits across nine different kinase groups, namely, AGC (protein kinase A, G and C group), atypical kinases (Hisk, BRD, PDHK), CAMK (Calcium/Calmodulin regulated kinases), CK1 (Casein Kinase 1 group), CMGC (CDK, MAPK, GSK3 and CLK kinases), STE (MAP kinase cascade kinases), TK (Tyrosine kinase), TKL (Tyrosine kinase-like group) and others **(** **Table 3** **)**. Highest number of transcripts was found to be associated with CMGC kinase group. Cytochrome P450s (CYPs) play an important role in physiology of fungi and are involved in biosynthesis of secondary metabolites and detoxification [70]. A total of 88 transcripts encoding CYP subfamily proteins were identified in *V. inaequalis* while selecting the unique hits from Pfam and InterproScan searches and BLASTp analysis against fungal Cytochrome P450 database **(File S10)**. Overall, this study had identified transcripts that might be involved in several important molecular and cellular functions of *V. inaequalis*.

**Table 3:** The number of genes encoding protein kinases in *V. inaequalis*.

Kinase group	No. of occurrences
AGC (protein kinase A, G and C group)	31
Atypical kinases (Hisk, BRD, PDHK)	35
CAMK(Calcium/Calmodulin regulated kinases)	33
CK1 (Casein Kinase 1 group)	14
CMGC (CDK, MAPK, GSK3 and CLK kinases)	58
STE (MAP kinase cascade kinases)	38
TK (Tyrosine kinase)	0
TKL (Tyrosine kinase-like group)	0
Other	31
Total	240

### Secretome of *V. inaequalis*

**Figure 5:**
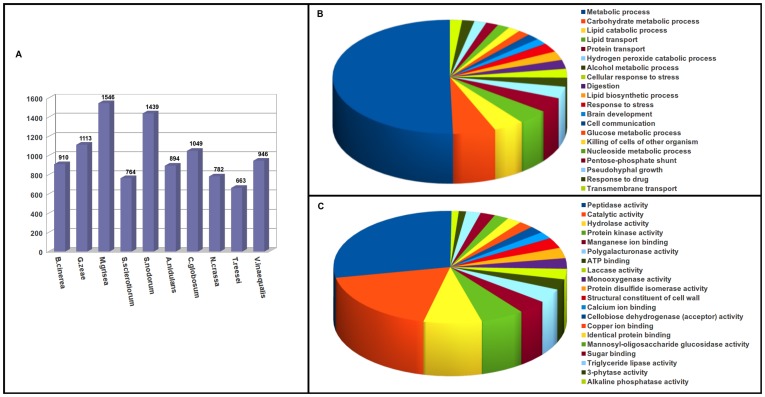
Secretome analysis of *V. inaequalis*. Comparative analysis of secretome sizes of various filamentous fungi (**A**), showing that the secretome size of *V. inaequalis* is comparable to other fungi. Gene Ontology (GO) term assignments to *V. inaequalis* secretome, based on significant GO slims, summarized into two main GO categories: biological process (**B**), and molecular function (**C**). Area under each pie represents the value in percent. Nearly 50% of genes are involved in metabolic processes as shown in **B**, while peptidase activity and catalytic activity are predominant molecular functions (**C**).

Secreted pathogenic proteins and effectors, better known as secretome are crucial for establishing infection on the host plant [71], [72]. These secreted proteins may disable plant defense and sabotage cellular processes to suit the needs of invading pathogens. There are a number of computational tools available that predict whether a protein is likely to be secreted or not [73]. We used SignalP [53]; to predict the presence of signal peptides and TMHMM [54]; to predict the presence of transmembrane helices to define the secretome of *V. inaequalis*. Those proteins which contain signal peptides but lack transmembrane helices are considered as secreted proteins. Following such criteria, 463 Set A transcripts and 483 Set B transcripts were predicted to be secreted (**Table 4**). Interestingly, *Venturia* seems to harbor similar number of secreted proteins to that of majority of phytopathogenic fungi (**Figure 5A**).

**Table 4:** Summary statistics for predicted secretome and RxLR effectors.

	Set A transcripts	Set B transcripts
Sequences >69 aa	13643	9115
Signal Cleavage site (+)	676	617
Transmembrane (−)	471	516
Candidate secretomes	463	483
Secretome with Pfam family	334	4
RXLR pattern present	28	17
Candidate RXLR effector (Stringent)	2	0
Candidate RXLR effector (Less stringent)	28	13

To functionally annotate the secretome, we performed Gene Ontology and Pfam protein domains searches. GO biological process analysis revealed that nearly 50% of Set A transcripts are involved in metabolism (**Figure 5B**). The overrepresentation of genes associated with metabolic processes has also been observed in the secretome of *Fusarium graminearum*, the causal organism of Fusarium Ear Blight disease of small grain cereals [74], and in the secretome of *Phytophthora infestans*, the casual agent of tomato and potato late blight diseases [75]. Several genes associated with transport (protein, lipid etc), response to stress, pseudohyphal growth and killing of cells of other organisms were found present in the secretome. The secretome appeared enriched for peptidase activity (22.05%), catalytic activity (14.17%) and hydrolase activity (6.03%), as depicted by GO molecular function analysis (**Figure 5C**). Quite a few genes that might regulate signaling cascades were also observed in the identified secretome.

Several Pfam domains were noticed in amino acid sequences of the transcripts encoding predicted secreted proteins of *V. inaequalis* **(File S11)**. The FAD binding domain, cutinase, eukaryotic aspartyl protease, hydrophobic surface binding protein A, glycosyl hydrolases families and those related to cell wall degradation were among the most frequently found Pfam domains in such transcripts. A domain associated with chitin recognition protein (PF00187) was one such domain. Chitin-binding proteins are thought to protect the fungal cell wall from chitinases that are produced by host plants [76]. Another important Pfam domain found in *V. inaequalis* is generally associated with isochorismatase family proteins. Conversion of isochorismate to 2, 3-dihydroxybenzoate and pyruvate has been reported to be catalyzed by such enzymes. Also, they are involved in synthesis of anti-microbial compounds such as phenazine [77] and siderophore and enterobactin [78]. Although isochorismatases are present in many filamentous ascomycetes, they are known to be secreted only in phytopathogenic fungi. As isochorismate is a precursor of SA (Salicyclic Acid, an important plant hormone involved in plant defense and systemic acquired resistance); the phytopathogens might use isochorismatases to sequester SA accumulation. This in turn might attenuate the plant defense [79]. Another important Pfam domain identified in *Venturia* secretome is associated with inosine-uridine preferring nucleoside hydrolase enzyme. This enzyme is important for parasitic organisms, which are deficient in *de novo* synthesis of purines and are dependent on salvaging the host purine nucleosides [80]. Interestingly the purine auxotrophs of *V. inaequalis* were compromised for pathogenesis on apple [81], suggesting the importance of inosine-uridine preferring nucleoside hydrolase enzyme during pathogenesis of *Venturia* on apple.

### Cell wall degrading enzymes present in *V. inaequalis*

As mentioned above, several Pfam domains involved in cell wall degradation were present in the secretome. Phytopathogens have impressive arsenal of plant cell wall degrading enzymes (CWDE) which are secreted to depolymerize different constituents of plant cell wall and required for successful pathogenesis. *Magnaporthe grisea*, the casual agent of rice blast disease has 30 enzymes for cellulose degradation and 44 enzymes for hemicellulose degradation [82], [83]. The present study reports six cutinases (PF01083; methyl esterases that degrade cutin), two cellulases (glycosyl hydrolase family 5), three pectate lyases and two pectin acetyl esterases in the secretome. Also, a large number of β-glucosidase were found (n = 22) in the scab pathogen, followed by α-mannosidase (n = 25), polygalacturonase (n = 19). Four contigs with PF00295 domain (encoding Glycosyl hydrolase family 28) were predicted to be secreted. This domain is present in enzymes involved in pectin degradation. Ten genes belonging to family GH 61 were present in the transcriptome. It is interesting to note that members of this family protein act as factors that enhance the hydrolysis of lignocellulose [82]. Thus, several CWDEs encoding transcripts of *Venturia* were identified in this study and data is summarized in **File S12**. It would be interesting to explore their function during establishment of apple scab disease. Interestingly, CWDEs are thought to play critical role in *Venturia*-apple interactions and have been speculated to be required for host penetration and nutrient uptake from the plant [1].

### Identification of *V. inaequalis* effectors

Beside CWDEs, small molecular weight secreted proteins of phytopathogenic fungi are known to reprogram the host metabolism and prevent the execution of plant defense responses [84], [85], [86], [87], [88]. These small molecular weight secreted proteins are well known as effectors. However, during million years of co-evolution, the host plants have evolved strategies to recognize pathogenic effectors and mount resistance (*R*) gene mediated disease resistance, which is potent enough to combat disease [89]. Such effectors are known as avirulence factors, as their presence render pathogen avirulent. Several avirulence factors were previously predicted from *V. inaequalis* and some of them are known to be race determinants [1], [2], [90]. However, till now no such Avr factors had been molecularly identified or cloned. In an attempt, Bowen et al [17] have identified 16 putative effector proteins of *Venturia* and demonstrated that three of them are induced during *in planta* infection. Presence of all 16 predicted effector proteins were noticed in the *Venturia* transcriptome sequenced in this study. Our transcriptome data could also reflect the presence of few *Cladosporium fulvum (syn. Passalora fulva)* (model ascomycetes fungi, extensively used to identify and characterize fungal effectors) effectors in *Venturia* (**Table 5**). Notably amongst them is a homolog of ECP6; the LysM domain containing effector of *C. fulvum*, which plays a critical role in its virulence [91] (in Set B; **Table 5**).

**Table 5:** Candidate effector of *Venturia inaequalis*.

	Contig Id	Organism	Accession number	E-value
**Set A transcripts**	contig_60045_1	Vice 2 (*V.inaequalis*)	EB149687.1	9.00E-028
	contig_46291_1	Vice 3 (*V.inaequalis*)	EB149718.1	1.00E-104
	contig_44222_1	Vice 5 (*V.inaequalis*)	FJ621507.1	1.00E-102
	contig_40056_1	Vice 6 (*V.inaequalis*)	EB152934.1	9.00E-042
	contig_53176_1	Vice 10 (*V.inaequalis*)	FJ621508.1	1.00E-049
	contig_35844_1	Vice 11 (*V.inaequalis*)	FJ621509.1	8.00E-043
	contig_9544_1	Vice 16 (*V.inaequalis*)	FJ621514.1	7.00E-067
**Set B transcripts**	contig_27031_1	Vice 1 (*V.inaequalis*)	EB148895.1	1.00E-026
	contig_40559_3	Vice 4 (*V.inaequalis*)	EB149885.1	2.00E-015
	contig_55213_5	Vice 6 (*V.inaequalis*)	EB152934.1	1.00E-004
	contig_34615_1	Vice 7 (*V.inaequalis*)	EB153294.1	4.00E-015
	contig_39772_3	Vice 9 (*V.inaequalis*)	EB153319.1	2.00E-048
	contig_57585_1	Vice 14 (*V.inaequalis*)	FJ621512.1	9.00E-003
	contig_34949_6	CLA_7 (*Passalora fulva*)	EU730588.1	0.002
	contig_35312_2	CLA_10 (*C.fulvum*)	X98578.1	0.003

Conserved RxLR motifs, located within N-terminal 60 amino acids downstream of signal peptide cleavage sites are present in many oomycetes effector proteins [92]. The RxLR motif is thought to be required for host translocation of effectors. Recently, presence of RxLR motifs in *M. grisea* and some other ascomycetes fungi has also been reported. In order to explore whether *V. inaequalis* also possess such RxLR effectors, amino acid sequences of secretome were subjected to motif analysis. A total of 41 (28 in Set A and 13 in Set B) potential RxLR effectors were identified through these searches **(** **Table 4** **)**. However, under stringent condition, we could only get two RxLR effectors. This suggests that the RxLR motif might not be a useful motif for identifying candidate effectors from *Venturia*. This could also reflect that oomycetes and fungi, separated by several hundred million years of evolution, might use different mechanism for effector delivery. However, availability of genome sequence could provide better representation of RxLR effectors in *Venturia*. In order to obtain functional insights about the predicted secreted effectors with RxLR motifs, BLASTx analysis and GO annotations were performed for each of them and the results are summarized in **File S13**. Interestingly, BLAST analysis revealed that several of putative RxLR containing sequences demonstrate homology with cell wall degrading enzymes, such as beta-glucosidase/beta-1,4-glucosidase, extracellular exo-polygalacturonase, pectate lyase A etc. Functional characterization of these effectors could help us in understanding how they interfere with host machinery and assist in scab disease establishment. Also, it would be interesting to explore whether any of these predicted effectors may function as avirulence factor(s) that govern race specificity on differential cultivars of apple. Identification of such factors would be really helpful in resistant breeding programs for ensuring durable resistance.

### Pathogenicity associated genes of *V. inaequalis*

**Figure 6:**
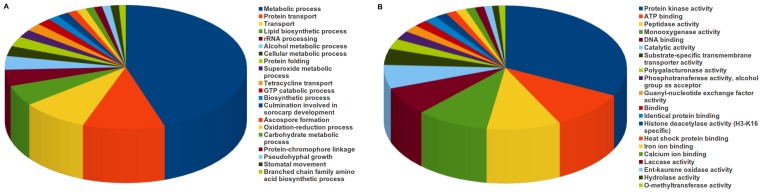
Gene Ontology classification of *V. inaequalis* orthologs of PHI genes. GO term assignments to *V. inaequalis* unigenes, with homology to PHI genes. **A:** represents biological process and **B:** represents the molecular function. PHI analysis revealed that the metabolic processes are overrepresented in PHI genes ortholog of *V. inaequalis*, thus highlighting the potential role of these processes in pathogenicity.

**Figure 7:**
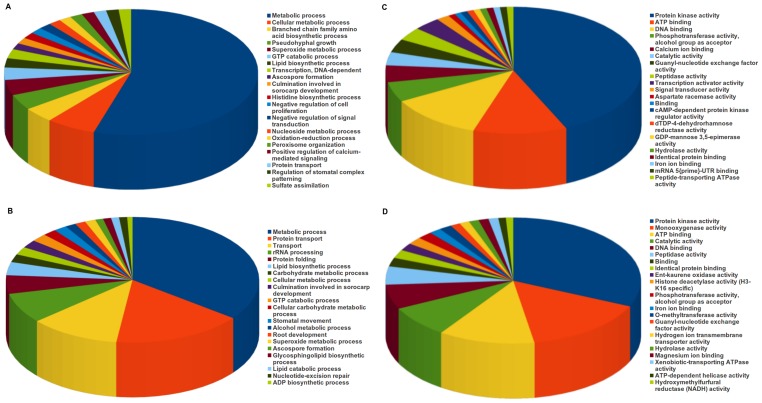
Gene Ontology classification of *V. inaequalis* orthologs of PHI genes that are associated with loss of pathogenicity and reduced virulence. GO term assignments to *V. inaequalis* unigenes with homology to PHI genes associated with loss of pathogenicity (**A**, **C**) and reduced virulence (**B**, **D**). **A**, **B** and **C**, **D** represents the biological process and molecular function, respectively. This analysis revealed that the majority of PHI genes associated with loss of pathogenicity and reduced virulence are involved in metabolic processes, thus highlighting the potential role of these processes in pathogenicity.

To predict potential pathogenicity genes of *V. inaequalis*, whole transcriptome BLASTp analysis against the pathogen-host interaction gene database (PHI database) was performed. PHI database is a collection of experimentally verified pathogenicity, virulence, lethal and effector genes from fungi, oomycetes and bacteria. The analysis resulted in 2,159 hits with E-value cut off of 1e^−05^. A total of 482 unique hits corresponding to known pathogenicity determinants of various pathogenic fungi (**File S14**), 102 hits corresponding to genes associated with loss of pathogenicity, 282 hits for reduced virulence and three for pathogenic effectors were observed in PHI database **(File S14)**. GO biological process analysis revealed that majority of *Venturia* transcripts were orthologous to PHI genes involved in metabolic processes, followed by those responsible for transport of proteins/lipids (**Figure 6A** **, File S15**). GO analysis further reflected that majority of transcripts orthologous to PHI genes associated with loss of pathogenicity (**Figure 7A**) and reduced virulence (**Figure 7B**), might also be involved in metabolic process. Surprisingly, holomogs for known effectors in PHI database largely correspond to metabolic process (**File S15**); suggesting *Venturia* effectors might be targeting host metabolic processes for successful colonization. The protein kinase activity, followed by ATP binding and peptidase activity were predominant GO molecular function (**Figure 6B** **, File S15**). Similar trend was also observed when transcripts with loss of pathogenicity (**Figure 7C**) and reduced virulence (**Figure 7D**) were analyzed for GO molecular function. This highlights the importance of energy dependent signaling cascades during establishment of apple scab disease.

Apart from transcripts having homologies within PHI database, the orthologs of other genes that are known pathogenicity determinants were also identified in this study. Notably, two contigs showing homology with hydrophobins were identified. Considering the fact that hydrophobins are required for conidial development, viability and pathogenic development of *M. grisea* [93], [94] as well as are involved in mediating fungal interaction with hydrophobic surfaces [95], it is encouraging to speculate that they might play role during apple scab pathogenesis.

### Comparative analysis of protein families of *V. inaequalis*

**Figure 8:**
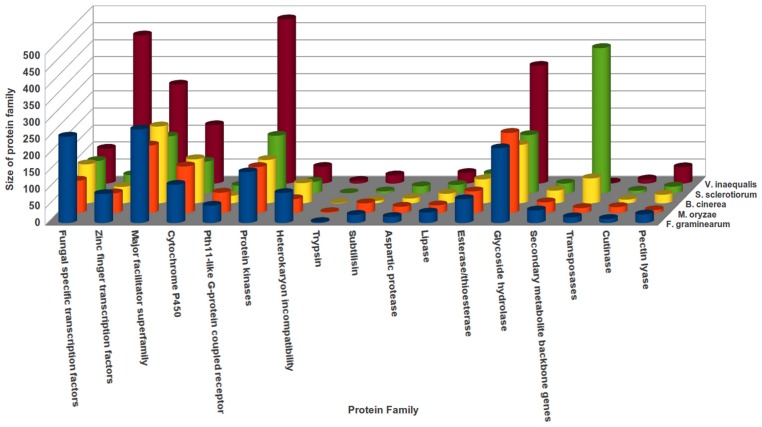
Comparative analysis of protein families of *V. inaequalis* with different plant fungal-pathogens. The histogram reflects the number of selected protein families across different phytopathogens namely *F. graminearum*, *M. oryzae*, *B. cinerea* and *S. sclerotiorum*.

The size of various protein families of *V. inaequalis* were compared with that of four plant pathogens; *F. graminearum*, *M. oryzae*, *B. cinerea* and *S. sclerotiorum* [52] **(File S16,** **Figure 8** **)**. The size of several protein families, such as cutinases (n = 12), glycoside hydrolase (n = 345) and cytochrome P450 (n = 171) were quite comparable across all these pathogens. However, the transposases (n = 3) were underrepresented while zinc finger transcription factors (n = 434) and protein kinases (n = 481) were found to be overrepresented in *V. inaequalis*. In general, overall number of glycoside hydrolases (GH) possessed by *V. inaequalis* (n = 238) is similar to that encoded by *M. oryzae* (n = 236), *B. fuckeliana* (n = 237), *A. nidulans* (n = 246), *A. oryzae* (n = 296), and *N. crassa* (n = 176) (**File S17**). Also the percentage representation of individual members of different GH family was quite comparable across different pathogens analyzed. However, overrepresentation of a few GH families was observed in *Venturia*. Notably, amongst them are GH1, GH17, GH31, GH38, GH47 and GH63 (**File S17**). Interestingly, a few GH families (GH13 and GH18) were underrepresented in *Venturia* as compared to other fungal pathogens. Also, few GH family members, generally present in other fungal pathogens, were found to be absent in *V. inaequalis* transcripts. We identified a few members of GH77 in *Venturia* which are otherwise found to be absent in other fungal pathogens analyzed in this study. The differences between protein families might point towards the underlying variability in pathogenicity mechanisms of these phytopathogens. The availability of *Venturia* genome sequence and multi-stage transcriptome data may facilitate further validation of this comparative study.

**Figure 9:**
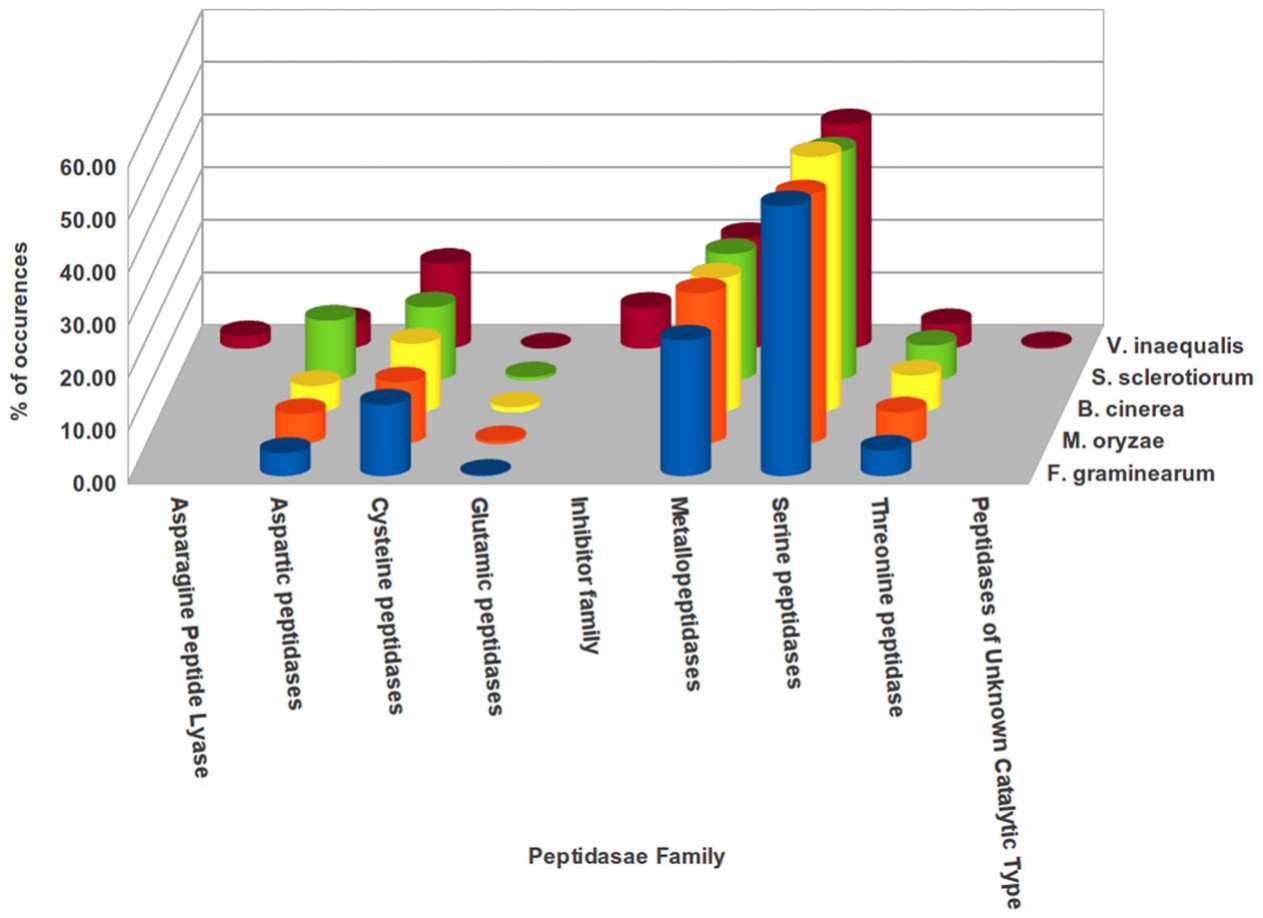
Histogram showing the abundance of Peptidase families in different plant fungal pathogens. *V. inaequalis* transcripts encoding peptidase families were predicted using MEROPS database and the respective percent sizes were compared with that of *F. graminearum*, *M. oryzae*, *B. cinerea* and *S. sclerotiorum*.

Search against MEROPS peptidase database led to identification of eight major categories of peptidases in *Venturia* transcriptome. **Figure 9** provides the list of peptidase family present in *Venturia* and along with their comparative account across different fungal pathogens. Serine peptidase, metallopeptidases and cysteine peptidases constitute the majority of peptidase family present in different fungal pathogens analyzed, including *V. inaequalis*. Interestingly though, the asparagine peptide lyase, inhibitors family and peptidases of unknown catalytic type were exclusively present in *V. inaequalis* **(File S18)**. We further attempted to identify the transcripts encoding various transporters in *Venturia*. **Table 6** represents the list of seven superfamilies of transporters present and also provides comparative enlisting of different subclasses present in different fungal pathogens. It is noteworthy that all these seven transporters superfamily involved in various physiological activities are overrepresented in *Venturia*, as compared to other fungal pathogens. The finding that transporters are overrepresented in *Venturia*, suggests that some of the transporters might be facilitating *Venturia* to obtain nutrients from the host during *in-planta* growth and might also assist it to tackle the altered pH homeostasis, generally observed during execution of plant defense. Also, the transporters might be assisting the apple scab pathogen to generate resistance against commonly used fungicides, as seen in case of *P. tritici-repentis* a wheat pathogen, wherein the efflux transporters are known to impart fungicide resistance [96].

**Table 6:** Percentage size of genes encoding membrane transporters in plant pathogens.

Superfamilies	TC No.	Subclass description	VI^a^	FG^a^	MO^a^	BC^a^	SS^a^
**Channels/Pores**	1.A	β-Type channels	223 (12.79%)	22 (3.33%)	23 (4.63%)	28 (5.42%)	21 (4.53%)
	1.B	α-Barrel porins	24 (1.38%)	1 (0.15%)	1 (0.2%)	1 (0.19%)	3 (0.65%)
	1.C	Pore-forming toxins(proteins and peptides)	1 (0.06%)	1 (0.15%)	0 (0.00%)	0 (0.00%)	0 (0.00%)
	1.D	Nonribosomally synthesized channels	0 (0.00%)	0 (0.00%)	0 (0.00%)	0 (0.00%)	0 (0.00%)
	1.E	Holins	0 (0.00%)	0 (0.00%)	0 (0.00%)	0 (0.00%)	0 (0.00%)
	1.F	Vesicle Fusion Pores	4 (0.23%)	1 (0.15%)	1 (0.2%)	2 (0.39%)	1 (0.22%)
	1.G	Viral Fusion Pores	0 (0.00%)	0 (0.00%)	0 (0.00%)	0 (0.00%)	0 (0.00%)
	1.H	Paracellular channels	0 (0.00%)	0 (0.00%)	0 (0.00%)	0 (0.00%)	0 (0.00%)
**Electrochemical potential-driven transporters**	2.A	Porters (uniporters, symporters, antiporters)	679 (38.93%)	405 (61.27%)	262(52.72%)	271(52.42%)	251 (54.09%)
	2.B	Nonribosomally synthesized porters	0 (0.00%)	0 (0.00%)	0 (0.00%)	0 (0.00%)	0 (0.00%)
	2.C	Ion-gradient-driven energizers	0 (0.00%)	0 (0.00%)	0 (0.00%)	0 (0.00%)	0 (0.00%)
**Primary active transporters**	3.A	P–P-bond-hydrolysis-driven transporters	436 (25.00%)	166 (25.11%)	151 (30.38%)	153 (29.59%)	138 (29.74%)
	3.B	Decarboxylation-driven transporters	4 (0.23%)	1 (0.15%)	2 (0.4%)	3 (0.58%)	2 (0.43%)
	3.C	Methyl-transfer-driven transporters	0 (0.00%)	0 (0.00%)	0 (0.00%)	0 (0.00%)	0 (0.00%)
	3.D	Oxidoreduction-driven transporters	105 (6.02%)	1 (0.15%)	1 (0.2%)	1 (0.19%)	1 (0.22%)
	3.E	Light absorption-driven transporters	10 (0.57%)	2 (0.3%)	1 (0.2%)	1 (0.19%)	1 (0.22%)
**Group translocators**	4.A	Phosphotransfer-driven group translocators	0 (0.00%)	0 (0.00%)	0 (0.00%)	0 (0.00%)	0 (0.00%)
	4.B	Nicotinamide ribonucleoside uptake permeases	0 (0.00%)	0 (0.00%)	0 (0.00%)	0 (0.00%)	0 (0.00%)
	4.C	Acyl CoA ligase-coupled transporters	38 (2.18%)	13 (1.97%)	13 (2.62%)	11 (2.13%)	10 (2.16%)
**Transmembrane electron carriers**	5.A	Transmembrane two-electron transfer carriers	2 (0.11%)	1 (0.15%)	1 (0.2%)	1 (0.19%)	1 (0.22%)
	5.B	Transmembrane one-electron transfer carriers	47 (2.69%)	3 (0.45%)	2 (0.40%)	1 (0.19%)	2 (0.43%)
**Accessory electron carriers**	8.A	Auxiliary transport proteins	43 (2.47%)	15 (2.27%)	10 (2.01%)	17 (3.29%)	10 (2.16%)
	8.B	Ribosomally synthesized protein/peptide toxins that target channels and carriers	0 (0.00%)	0 (0.00%)	0 (0.00%)	0 (0.00%)	0 (0.00%)
	8.C	Non-ribosomally synthesized toxins that target channels and carriers	0 (0.00%)	0 (0.00%)	0 (0.00%)	0 (0.00%)	0 (0.00%)
**Incompletely characterized transporters**	9.A	Recognized transporters of unknown biochemical mechanism	91 (5.22%)	23 (3.48%)	23 (4.63%)	23 (4.45%)	20 (4.31%)
	9.B	Putative uncharacterized transport proteins	32 (1.83%)	6 (0.91%)	6 (1.21%)	4 (0.77%)	3 (0.65%)
	9.C	Functionally characterized transporters lacking identified sequences	1 (0.06%)	0 (0.00%)	0 (0.00%)	0 (0.00%)	0 (0.00%)

a : Number of genes/transcripts encoding transporters were identified for each pathogen and the percentage of each subcategories of transporters are provided in bracket. VI: *V. inaequalis*, FG: *F. graminearum*, MO: *M. oryzae*, BC: *B. cinerea* and SS: *S. sclerotiorum*.

### Phylogenomic analysis of *V. inaequalis*

**Figure 10:**
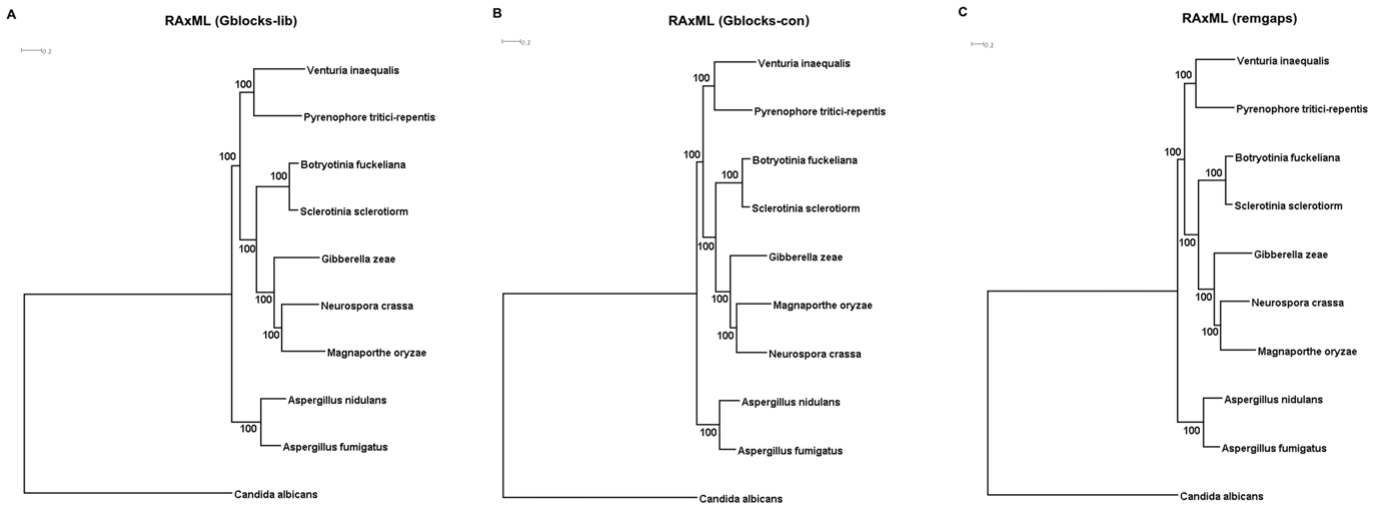
Phylogenomic analysis of *V. inaequalis*. Maximum Likelihood based phylogenetic analysis using RAxML tool, on three different super-alignments of *V. inaequalis* and that of other nine selected fungal pathogens, were used to establish phylogenomic relationship. **A, B, C** represents phylogenetic tree from super-alignment constructed by Gblocks-con, Gblocks-lib and remgaps, respectively using Hal pipeline.

The reference amino acid sequences of nine organisms (**File S19**), *Aspergillus fumigatus*, *Aspergillus nidulans*, *Candida albicans*, *Magnaporthe oryzae*, *Neurospora crassa*, *Sclerotinia sclerotiorum*, *Pyrenophora tritici-repentis*, *Gibberella zeae*, *Botryotinia fuckeliana* and assembled sequences of *V. inaequalis* obtained in this study, were analyzed using Hal, an automated pipeline for phylogenetic analysis of genomic protein sequence data [58]. The Hal pipeline generated three separate sets of alignments, one removing all gap-containing columns (remgaps) and two removing problematic regions of alignments based on the default conservative (Gblocks-con) and liberal (Gblocks-lib) options of the program Gblocks [61]. Three phylogenetic trees representing three super-alignments (remgaps, Gblocks-con and Gblocks-lib) were generated and they were found to be well resolved and robust with high bootstrap values for different clades (**Figure 10**). As expected, different clades were observed in the phylogenetic trees and relationship between most of the pathogens analyzed were similar to that observed by Gao et al [97]. Interestingly, in every phylogentic tree, each generated by different methods, *V. inaequalis* was placed closer to *P. tritici-repentis* (**Figure 10**). As *V. inaequalis* and *P. tritici-repentis* are placed in Pleosporaceae family, our study strengthens the classification of *V. inaequalis* into this family and establishes its closeness to that of other members of Pleosporaceae family at genome wide scale.

## Conclusion

In this study, we report *de novo* assembly of the transcriptome of *V. inaequalis*, the apple scab pathogen, and its in-depth computational analysis to explore genome wide insights about this deadly pathogen. In total, we obtained 94,350,055 SE reads specific to *Venturia*, assembled them into 62,061 contigs. Out of which 29,750 transcripts demonstrated significant similarity to sequences in other species by BLAST analysis, representing a total of 24,571 unique genes (Set A). No significant homolog was found for 32,311 assembled transcript sequences (Set B). Functional annotations using GO, EC, KEGG, InterproScan, Pfam analysis and searches against various databases identified genes that might be participating in several important biological and metabolic pathways. A total of 463 Set A transcripts and 483 Set B transcripts were found encoding putative secreted proteins. Metabolism was the predominant biological process ontology category while peptidase, catalytic and hydrolase activities were amongst predominant molecular function categories. Also the present work identified several cell wall degrading enzymes and Pfam domains participating in cell wall degradation from *Venturia* transcriptome. Furthermore, a few host translocated putative RxLR effectors, orthologs of several known candidate effectors and those showing homologies to known pathogenicity determinants of various pathogenic fungi as per PHI database entries were identified in this study. A large number of transcripts encoding membrane transporters were identified and the comparative analysis unraveled that the number of transporters encoded by *Venturia* are significantly more as compared to that encoded by several other important plant fungal pathogens. Overall, the transcriptome analysis of *V. inaequalis* provided wealth of information which would facilitate further research to understand the biology and pathogenicity mechanism of this pathogen, which in turn would make possible to evolve novel strategies for engineering disease resistance in apple.

## Supporting Information

File S1 155 experimentally validated nucleotide sequences for *V. inaequalis* available at GenBank.
(TXT)

File S2 Sequences of known effectors of various pathogenic fungi.
(TXT)

File S3 Sequences of known *V. inaequalis* effectors.
(TXT)

File S4 Effect of k-mer size on assembling performance of transcriptome using Velvet, SOAPdenovo and ABySS.
(XLS)

File S5 Dissimilar sequence groupings for assembled *V. inaequalis* transcriptome sequences.
(XLS)

File S6 Set A transcripts sequences.
(ZIP)

File S7 Set B transcripts sequences.
(ZIP)

File S8 Assembly validation with known *V. inaequalis* nucleotide sequences.
(XLS)

File S9 Assembly validation through Sanger Sequencing.
(DOC)

File S10 Blast analysis result against Fungal Cytochrome P450 database.
(XLS)

File S11 Pfam domains predicted to be present in the secretome of *V. inaequalis*.
(XLS)

File S12 Cell wall degrading enzymes predicted in transcriptome of *V. inaequalis*.
(XLS)

File S13 Candidate RxLR effector summary.
(XLS)

File S14 Summary of PHI database gene orthologs in *V. inaequalis*.
(DOC)

File S15 GO annotation of PHI gene orthologs of *V. inaequalis*.
(XLS)

File S16 Sizes of selected protein families in *V. inaequalis* and other plant pathogens.
(XLS)

File S17 Percentage of carbohydrate-degrading enzymes in ascomycetes fungal pathogens, arranged by GH family.
(XLS)

File S18 Protease genes in different fungal genomes, arranged by MEROPS family.
(XLS)

File S19 Number of reference protein sequences of selected phytopathogenic fungi used for Phylogenomics.
(DOC)
