# Supplementary material for: De Novo Transcriptome Sequencing and Analysis for Venturia inaequalis, the Devastating Apple Scab Pathogen
Source: PLoS One. 2013 Jan 17;8(1):e53937. doi: 10.1371/journal.pone.0053937 (PMC3547962; doi:10.1371/journal.pone.0053937)
Supplement: File S9 — Assembly validation through Sanger Sequencing. (DOC) [file pone.0053937.s009.doc]

**File S9:** Assembly validation through Sanger Sequencing

| **S.No.** | ***De novo* assembled Contigs** | | **Sequences Obtained through Sanger Method** | | | |
| --- | --- | --- | --- | --- | --- | --- |
|  |  |  |  | **BLAST Analysis*** | | |
|  | **Name** | **Length** | **Length** | **Score** | **E-Value** | **Identity** |
| 1 | Contig_8721 | 251 | 131 | 239 | 3e-68 | 99% |
| 2 | Contig_26462 | 1035 | 184 | 343 | 1e-98 | 100% |
| 3 | Contig_27327 | 1856 | 217 | 385 | 4e-111 | 99% |
| 4 | Conitg_31168 | 979 | 122 | 154 | 4e-73 | 96% |
| 5 | Contig_35313 | 2517 | 162 | 292 | 3e-83 | 99% |
| 6 | Contig_40770 | 3636 | 242 | 431 | 1e-124 | 98% |
| 7 | Contig_50883 | 1962 | 207 | 374 | 1e-107 | 99% |
| 8 | Contig_54127 | 2874 | 184 | 315 | 8e-90 | 97% |
| 9 | Contig_57771 | 1431 | 145 | 259 | 2e-73 | 99% |
| 10 | Contig_58791 | 1890 | 871 | 1592 | 0.0 | 99% |
| 11 | Contig_59687 | 747 | 674 | 1202 | 0.0 | 99% |
| 12 | Contig_60279 | 993 | 211 | 381 | 3e-110 | 99% |
| 13 | Contig_61863 | 558 | 179 | 322 | 8e-93 | 99% |
| 14 | Contig_31803 | 1810 | 1172 | 2165 | 0.0 | 100% |
| 15 | Contig_42320 | 1895 | 443 | 573 | 3e-167 | 99% |

*BLAST2 analysis of sequences obtained from Sanger method with those obtained from *GAIIx*
