# Supplementary material for: De Novo Transcriptome Sequencing and Analysis for Venturia inaequalis, the Devastating Apple Scab Pathogen
Source: PLoS One. 2013 Jan 17;8(1):e53937. doi: 10.1371/journal.pone.0053937 (PMC3547962; doi:10.1371/journal.pone.0053937)
Supplement: File S14 — Summary of PHI database gene orthologs in V. inaequalis. (DOC) [file pone.0053937.s014.doc]

**Table S14: Summary of PHI database gene orthologs in *V. inaequalis***

| **Phenotypic category** | **Query hit count** | **Unique PHI number** |
| --- | --- | --- |
| Loss of Pathogenicity | 416 | 102 |
| Reduced virulence | 1250 | 282 |
| Effector (plant avirulence determinant) | 25 | 3 |
| Lethal | 7 | 2 |
| Increased virulence (Hypervirulence) | 29 | 8 |
| Chemistry target – phenotype unknown | 2 | 2 |
| Enhanced antagonism | 1 | 1 |
| Resistant to chemical | 37 | 13 |
| Sensitive to chemical | 1 | 1 |
| Unaffected pathogenicity | 388 | 66 |
| Wild-type mutualism | 1 | 1 |
| N/A | 2 | 1 |
