# Supplementary material for: De Novo Transcriptome Sequencing and Analysis for Venturia inaequalis, the Devastating Apple Scab Pathogen
Source: PLoS One. 2013 Jan 17;8(1):e53937. doi: 10.1371/journal.pone.0053937 (PMC3547962; doi:10.1371/journal.pone.0053937)
Supplement: File S19 — Number of reference protein sequences of selected phytopathogenic fungi used for Phylogenomics. (DOC) [file pone.0053937.s019.doc]

**Table S19**: **Number of reference protein sequences of selected phytopathogenic fungi used for Phylogenomics**

| **Organism name** | **Sequence count** |
| --- | --- |
| *Aspergillus fumigatus* | 9649 |
| *Aspergillus nidulans* | 9541 |
| *Candida albicans* | 14633 |
| *Magnaporthe oryzae* | 14010 |
| *Neurospora crassa* | 9844 |
| *Sclerotinia sclerotiorum* | 14446 |
| *Pyrenophora tritici-repentis* | 12169 |
| *Gibberella zeae* | 11700 |
| *Botryotinia fuckeliana* | 16389 |
| *Venturia inaequalis* | 4701 |
